# The Resilience and Change in the Biocultural Heritage of Wild Greens Foraging Among the Arbëreshë Communities in Argolis and Corinthia Areas, Peloponnese, Greece

**DOI:** 10.3390/plants14213371

**Published:** 2025-11-04

**Authors:** Mousaab Alrhmoun, Naji Sulaiman, Ani Bajrami, Avni Hajdari, Andrea Pieroni, Renata Sõukand

**Affiliations:** 1 University of Gastronomic Sciences, Piazza Vittorio Emanuele II 9, 12042 Pollenzo, Italy; n.sulaiman@unisg.it; 2 Museum of Natural Sciences “Sabiha Kasimati”, University of Tirana, Sheshi Nënë Tereza 4, 1010 Tiranë, Albania; ani.bajrami@fshn.edu.al; 3 Department of Biology, Faculty of Mathematical and Natural Science, University of Prishtina, Mother Theresa St., 10000 Prishtinë, Kosovo; avni.hajdari@uni-pr.edu; 4 Department of Medical Analysis, Tishk International University, Erbil 4001, Iraq; 5 Department of Environmental Sciences, Informatics and Statistics, Ca’ Foscari University of Venice, Via Torino 155, 30172 Venezia, Italy; renata.soukand@unive.it

**Keywords:** ethnobotany, wild greens, Arbëreshë, Arvanites, Greece, local ecological knowledge

## Abstract

The transformation of Local Ecological Knowledge (LEK) among minority populations undergoing cultural and linguistic assimilation over time is poorly understood. Arbëreshë communities in Greece, who have preserved Albanian-derived traditions for centuries, offer a unique opportunity to examine how folk plant knowledge adapts over time. This study examines the linguistic labels and culinary uses of wild greens among Arbëreshë (or Arvanites), an ethno-linguistic minority traditionally speaking Arbërisht or Arvanitika, the Tosk dialect of Albanian, who have resided in the Argolis and Corinthia regions of the Peloponnese for several centuries. In 2025, fieldwork was conducted in four rural Arbëreshë villages in the Argolis and Corinthia regions of Greece, combining semi-structured interviews with 24 elderly participants, participant observation, and the collection and identification of botanical specimens. The contemporary dataset was compared with historical ethnobotanical records from the 1970s to assess temporal changes in the use of wild vegetables and folk plant nomenclature. Our results reveal that current Arbëreshë ethnobotanical heritage has undergone profound Hellenisation, with 62% of folk plant names of Greek origin, 14% Albanian, and 24% hybrid, reflecting strong linguistic and cultural assimilation over the past half-century. The traditional boiled green mix (*lakra* in Arbëreshë, *chorta* in Greek) remains central to the local cuisine, which is rooted in foraged plants, although its culinary applications have diversified. In total, 37 taxa of wild vegetables across 37 genera and 14 families were documented in 2025, compared with 21 taxa across 21 genera in the filtered 1970 dataset. Core families, such as Asteraceae and Brassicaceae, remained dominant, while new families, like Malvaceae and Portulacaceae, appeared, possibly indicating both ecological and culinary changes. These findings raise questions about whether the Arbëreshë wild vegetable heritage was strongly influenced by the surrounding Greek majority or primarily acquired after migration, potentially facilitated by intermarriages and shared Orthodox Christian affiliation. Overall, our study highlights a largely Hellenised Arbëreshë biocultural heritage and underscores the urgent need for national and regional stakeholders to recognise and celebrate the remaining minority’s linguistic and ethnobotanical diversity. The transformation of local ethnobotanical knowledge over the past fifty years appears influenced by ecological availability, socio-cultural dynamics, and changing taste preferences.

## 1. Introduction

The Albanian Arbëreshë, also known in Greece and occasionally in the literature as Arvanites, are an ethno-linguistic minority of Albanian origin who settled in mainland and insular Greece between the 14th and 16th centuries [1,2]. The relocation of Arbëreshë to Greece is linked to the depopulation of these regions due to continuous conflicts and internal wars, as well as to the imperial decree that required military forces to be stationed on Greek territory in the 14th century [1]. Their historical presence is reflected in local toponymy, customs, and oral traditions [2,3,4]. Over time, however, most Arbëreshë communities in mainland Greece have undergone a process of progressive Hellenization, particularly since the formation of the modern Greek state in the 19th century [5]. This process, shaped by national integration policies and sociolinguistic dynamics, has led to a significant decline in the use of the Arvanitika dialect and the assimilation of cultural practices into the dominant Greek framework [2,6,7]. This process intensified during the 19th and 20th centuries, marked by cultural assimilation, the marginalisation of minority identities, and the near disappearance of Arvanitika, the Tosk dialect of the Albanian language [8,9].

In contrast to their counterparts in Southern Italy, where the Arbëreshë (the Albanian diaspora in Southern Italy, which includes many who migrated from Albania via Greece in the 15th century) have had their identity more actively preserved and institutionally supported [8,10], the Arbëreshë in mainland Greece followed a different path. The combined forces of national integration policies, marginalisation and stigmatisation of linguistic differences, and rural-to-urban migration have dramatically reduced both the use of Arvanitika and the intergenerational transmission of cultural knowledge [6,11,12,13]. Immigration, particularly during the post-war decades, further accelerated this transformation: as younger generations left rural areas in search of work or education in cities, local dialects, subsistence practices, and place-based knowledge systems weakened [14,15].

Simultaneously, land use changes, including the abandonment of traditional agricultural terraces, the decline of small-scale herding and subsistence farming, and the expansion of monocultures and tourism-related development, transformed the ecological and social landscape of many villages [16,17,18]. As a result, the daily interaction with plants and natural environments that sustained ethnobotanical knowledge was disrupted. These environmental changes, combined with shifting economic and social structures, contributed to the erosion of orally transmitted ecological knowledge, especially among younger generations who were no longer tied to ancestral livelihoods or seasonal rhythms [15].

Nevertheless, in a few isolated mountain communities in the Argolis region of Central Greece, notably Limnes, Arachneo, Aggelokastro, and Sofiko, elderly speakers continue to retain fragments of the Arbëreshë language and aspects of traditional ecological knowledge [7]. These villages, geographically removed from urban centres and located in rugged terrain, have maintained a certain degree of cultural autonomy, making them valuable sites for investigating residual ethnobotanical heritage.

Ethnobotanical knowledge, also known as local ecological knowledge (LEK), is typically transmitted orally and is strongly tied to both the landscape and cultural identity [18,19]. Several studies have highlighted the nexus between diaspora communities and their adapted (and travelled) local ecological knowledge [20,21]. A growing body of research highlights the link between linguistic and ecological diversity [19] and the fact that language loss is often accompanied by the erosion of environmental memory [22]. Yet some scholars have shown that foraging knowledge and plant uses can persist even when the original linguistic categories collapse, surviving in embodied practices and everyday use. In contrast, folk plant names may be retained, translated, or lost [21,23,24]. As such, the study of folk plant nomenclature provides a powerful lens through which to examine the relationship between language attrition, cultural resilience, and biocultural memory. In this context, ethnobotanical terminology among Arbëreshë speakers in Argolis offers a compelling case study. By examining the preservation, replacement, or loss of plant names of Albanian origin, it becomes possible to trace broader processes of cultural change and continuity, particularly in relation to migration, land abandonment, and social transformation.

This study aimed to: (1) document wild greens currently known by the Albanian-speaking communities in Argolis and Corinthia, Peloponnese, Greece; (2) assess changes in the diversity of wild greens used, by comparing the documented contemporary ethnobotanical data with a historical dataset from the 1970s; (3) evaluate the resilience and transformation of folk plant nomenclature, focusing on the retention, replacement, or hybridization of Albanian-origin names; and (4) examine how socio-cultural and ecological changes, including language shift and altered land use practices, may have influenced the continuity and adaptation of local ecological knowledge in these communities.

## 2. Materials and Methods

### 2.1. Study Area and Data Collection

**Figure 1:**
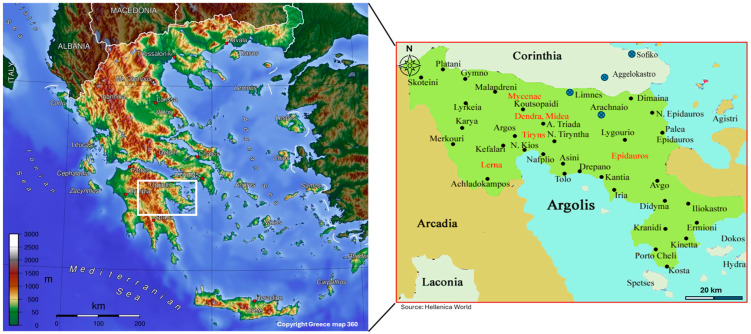
Study area map within the map of Greece. Locations of the villages where the study was conducted (blue circles and crosses refer to the studied villages of Limnes, Arachneo, Aggelokastro, and Sofiko) in the Argolis and Corinthia regions of Greece. Copyright Information: GreeceMap360: © 2025 Newebcreations. All rights reserved. Greece Map 360. Available online: https://greecemap360.com/ (accessed on 8 October 2025). Hellenica World: © Foundation of the Hellenic World. All rights reserved. Available online: https://www.hellenicaworld.com/ (accessed on 8 October 2025).

The study was conducted in the Argolis region of the northeastern Peloponnese, Central Greece. Fieldwork focused on four villages: Limnes, Arachneo, Aggelokastro, and Sofiko, known for their historical Albanian populations (Figure 1). These villages are located in hilly and semi-mountainous terrain, ranging from 250 to 800 m above sea level. A Mediterranean climate with mixed land uses, including small-scale farming (i.e., olive groves), and areas of secondary vegetation, characterises them. Due to their relative geographic isolation from urban and coastal centres, these communities have preserved residual elements of Albanian cultural and linguistic heritage, particularly among the elderly.

Over the past decades, however, the region has experienced significant demographic and land use changes, including population decline due to rural outmigration, abandonment of traditional agriculture, and ecological succession in previously cultivated or grazed areas. These transformations have influenced both the availability of wild plant resources and the transmission of local environmental knowledge. Between March and May 2025, ethnobotanical data were collected through semi-structured interviews with 24 elderly participants (16 men and eight women, aged 64–83) who identified themselves as native or heritage Albanian speakers. Such knowledgeable individuals, especially those familiar with local botanical plants, were increasingly rare to find in these villages. Interviews were conducted in Greek and, where possible, in Arvanitika dialect fragments, depending on speaker fluency. Participants were asked to list and describe the wild vegetables traditionally used for food and foraged in the surrounding landscape. For each plant mentioned, the following data were recorded local folk name(s) (with special attention to names of Albanian origin), used parts (e.g., leaves, shoots, bulbs), culinary preparation (e.g., boiling, salad, seasoning), and quotation frequency (classified as R = rarely, C = commonly, VC = very commonly). The Code of Ethics of the International Society of Ethnobiology was adhered to during this research, and every interviewee was informed about the aims of this study in advance [24]. Voucher specimens were collected and identified using the standard reference of the Greek Flora [25], verified using the online annotated checklist of the Greek flora (https://portal.cybertaxonomy.org/flora-greece/intro, accessed on 8 September 2025) and deposited at the Herbarium of the Bio-Cultural Diversity Lab of the Department of Environmental Sciences, Informatics, and Statistics, Ca’ Foscari University of Venice, Italy. Botanical nomenclature was aligned with the World Flora Online database [26]. Recorded folk names were transcribed in the Latin alphabet, following standard rules of Albanian and (Latinised) Greek orthographies.

Folk plant names of likely Albanian etymology were highlighted and later cross-checked with a comparative Albanian-language botanical lexicon [27], and regional studies to determine their origins. Special attention was given to whether these names coexisted with Greek alternatives, had been entirely replaced, or were still actively used.

### 2.2. Historical Reference Dataset (1970)

For comparative purposes, a dataset from the US anthropologist Mary Clark Forbes, who conducted extensive field studies in 1970 in Eastern Argolis, was used as a historical reference for the foraged flora of Argolis. Although the original research was rooted in deep ethnography and ethnobotanical in its essence, it did not record folk plant names, and, nevertheless, still provides a valuable list of wild or semi-wild plant taxa used for food in the same region at that time.

The 1970 list includes 38 plant taxa, primarily recorded at the genus level, along with brief notes on edible parts. While it is not certain that Forbes’ participants were of Albanian origin, the data were collected from eastern Argolis localities, thus offering a relevant baseline for temporal comparison of plant uses at the genus level.

To ensure the validity of diachronic comparison, we critically assessed the comparability of our 2025 dataset with the 1970 dataset by Clark Forbes. While both studies were conducted in the Argolis region in the spring season and documented wild edible plants, there are some limitations. First, although Forbes conducted her study in the eastern Argolis area, and thus geographically, there is no perfect overlap with our field sites. Second, sample sizes are not comparable as Forbes did not report participant numbers, whereas our 2025 study involved 24 informants.

Given these caveats, comparability was reasonable at the taxonomic level of plant genera, as the 1970 study did not collect botanical vouchers, and neither Forbes recorded vernacular plant names. We therefore limited the comparative analysis to the presence, absence, and innovation of wild green plant genera, while linguistic analysis was restricted to the 2025 dataset. By explicitly acknowledging these limitations, we sought to strike a robust balance between the attempt at a diachronic comparison and methodological reproducibility.

### 2.3. Data Analysis

Data were processed and analysed using R version 4.4.2 and SAS 9.4. The analysis focused on three primary objectives: describing the structure of the 2025 dataset, comparing it temporally with historical data from 1970, and assessing the degree of linguistic resilience in the preservation of folk plant nomenclature.

First, descriptive statistics were used to quantify the total number of plant taxa recorded in 2025 and to calculate the number and percentage of taxa bearing folk names of Albanian origin. The distribution of folk names was examined across botanical families and culinary preparation methods (e.g., boiled greens, salads, and seasonings). Citation frequencies were classified as rare, R, if wild green was quoted by less than 10% of the informants; common, C, if mentioned by more than 10% and less than 40% of the informants; very common, VC, if quoted by more than 40% of the informants.

Second, a diachronic comparison of the taxonomic lists from 1970 and 2025 was conducted to evaluate changes in species composition and ethnobotanical practices over time. Since the 1970 dataset did not report vernacular plant names, our analysis of continuity focuses on the persistence of taxa rather than the names themselves. Thus, categories of continuity, loss, and innovation are applied strictly to the taxonomic level. Linguistic variation could only be assessed within the 2025 dataset, where multiple folk names were recorded for specific genera. Genera common to both datasets were identified, and changes were categorised into three types: (1) continuity, where a plant continued to be used; (2) loss, where the genus was not mentioned in the 2025 data; and (3) innovation, where new taxa appeared in the 2025 data but were absent from the 1970 record. This allowed us to assess not only linguistic shifts but also broader cultural and ecological transformations in plant use practices.

Third, a linguistic resilience assessment was carried out to evaluate the extent of retention of Albanian folk names. The proportion of Albanian-origin names was calculated as a percentage of the total number of folk plant names recorded in 2025. Patterns of linguistic replacement were explored by identifying cases where Greek names had supplanted Albanian terms. Additionally, we examined whether specific wild greens were more likely to retain Albanian names or undergo lexical replacement, highlighting the uneven impact of language shift across plant-use categories. Finally, a series of multivariate analyses was considered to explore the deeper structure in the dataset. Correspondence Analysis (CA) was proposed to group plants based on naming origin and usage frequency. In addition, contingency tables and Chi-square tests were suggested to assess associations between the frequency of wild plant quotations and the retention of Albanian terminology. These analytical tools provide insight into the interaction between cultural resilience and linguistic attrition, particularly within an ageing minority population undergoing rapid sociolinguistic change.

## 3. Results

### 3.1. Botanical Diversity Change and Culinary Traditions Among the Greek Albanians

The wild greens dataset from 1970 included 21 species belonging to 21 genera across 10 families, whereas the 2025 dataset comprised 37 species from 37 genera distributed among 14 families.

**Figure 2:**
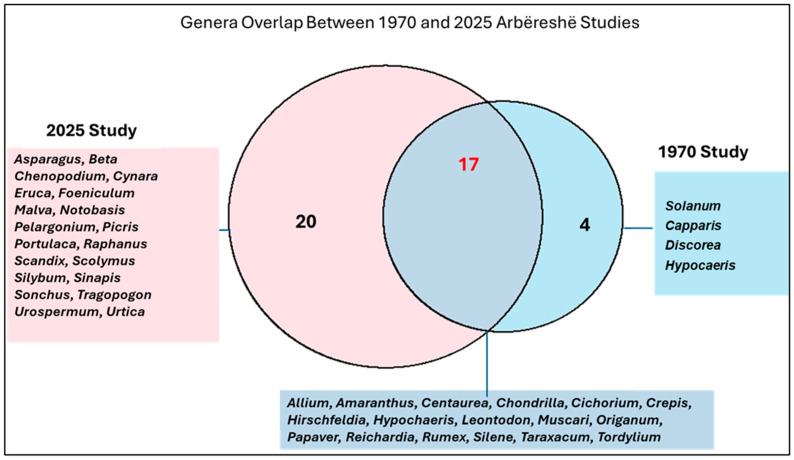
Overlap of the gathered and consumed wild greens genera between the current study and the field study conducted in Eastern Argolis in the 1970s.

Seventeen genera were consistently present in both 1970 and 2025, including *Allium, Amaranthus*, *Centaurea*, *Chondrilla*, *Cichorium*, *Origanum*, *Taraxacum*, and others, demonstrating continuity in core edible wild plant resources over time. Meanwhile, four genera recorded in 1970, such as *Solanum* (Solanaceae), *Capparis* (Caparaceae), *Discorea* (Dioscoreaceae), and *Hypochaeris* (Asteraceae), were absent in 2025. In contrast, twenty genera appeared exclusively in 2025, including *Asparagus* (Asparagaceae), *Beta* (Amaranthaceae), *Pelargonium* (Geraniaceae), *Raphanus* (Brassicaceae), and others (Figure 2), reflecting an expansion in both taxonomic diversity and ecological breadth of edible wild plants (Figure 2).

At the family level, the diversity of Asteraceae increased from nine to 14 genera, Brassicaceae from two to four, and Apiaceae from one to three, while Lamiaceae declined markedly from four genera to just one genus. Additionally, Fabaceae and Solanaceae, which were present in 1970, were absent from the 2025 dataset. These shifts highlight notable changes in family-level diversity over the 55-year interval (see Table 1).

The comparison of plant parts used in the two periods reveals both continuity and change in foraging practices and dietary preferences. In both studies, young aerial parts dominated the records, with genera such as *Amaranthus*, *Cichorium*, *Hirschfeldia*, *Reichardia*, *Rumex*, *Silene*, *Sonchus*, *Taraxacum*, and *Tordylium* consistently valued for their palatability, seasonal availability, and ease of preparation. The use of flowering structures increased slightly in 2025, with taxa such as *Origanum* (flowering tops) and *Cynara* (flower receptacles) appearing, replacing the 1970 record of *Matricaria* (flower heads). This change may indicate a refined appreciation for floral textures or an improved understanding of ethnobotanical documentation. Specific categories persisted, albeit with shifts in genera; for example, shoots of *Chondrilla*, *Rumex*, and *Tamus* were recorded in 1970, whereas *Asparagus*, *Silene*, and *Tragopogon* featured prominently in 2025.

The culinary uses documented in the 2025 study (Table 1) highlight the enduring centrality of boiled green mixes, locally known as *lakra,* which account for the majority of preparations (20 out of all recorded uses). This traditional method commonly involves taxa such as *Beta*, *Centaurea*, *Chenopodium*, *Cichorium*, *Chondrilla*, *Crepis*, *Hirschfeldia*, *Leontodon*, *Malva*, *Papaver*, *Picris*, *Raphanus*, *Reichardia*, *Scandix*, *Silene*, *Sinapis*, *Sonchus*, *Taraxacum*, *Tordylium*, *Tragopogon*, *Urospermum*, and *Urtica*, often combined to create nutritious and flavourful boiled green mixes. Other commonly reported uses, including simple snacks made from *Cynara*, *Notobasis*, and *Silybum*, as well as cooked dishes with *Asparagus*, *Muscari*, and *Scolymus*, and fresh salads featuring *Eruca*, *Portulaca*, *Reichardia*, and *Tragopogon,* emphasise the versatility of wild plants in everyday diets. The inclusion of seasonings derived from *Allium*, *Foeniculum*, *Origanum*, and *Pelargonium*, the latter also used in jams and sweet preserves, as well as more elaborate preparations such as dolmades (stuffed leaves) made with *Rumex*, reflects the role of wild taxa not only as sources of nourishment but also as essential flavour enhancers. Occasional mentions of pickling, particularly of *Muscari* bulbs, point to preservation practices that further extend their utility, while also mellowing the otherwise strong or bitter flavour of the bulbs. Collectively, these data reveal a rich, adaptable, and enduring culinary tradition rooted in the flexible use of a diverse array of wild plant species.

### 3.2. Ethnolinguistic Origins and Citation Frequency

In our field data (2025 study), out of the 37 wild plant taxa recorded, five (13.5%) were associated with folk names of pure Albanian origin, including *Beta vulgaris, Chenopodium album*, *Silene vulgaris*, *Sonchus oleraceus*, and *Urtica urens* (Table 2). The majority, 23 taxa (62.16%), were linked to Greek-origin names, underlining the strong historical and linguistic influence of Greek on the local folk botanical vocabulary. Locals also reported seven folk names, which were hybrid Albanian-Greek, representing 24.32% of all phytonyms recorded across the study area. The plants used by this group belong to the genera *Amaranthus*, *Allium, Cynara*, *Eruca, Malva*, *Papaver, Reichardia*, and *Tragopogon*.

In the 2025 dataset, wild plant taxa were grouped by frequency of quotation into three categories (Table 3). Commonly quoted taxa (C) represented 37.8% of all genera (14 out of 37), including culturally significant species such as *Origanum*, *Rumex*, and *Pleurotus*, which are well embedded in local foodways and valued for their versatility. Widespread reported taxa (VC) accounted for 32.4% (12 genera), such as *Crepis* (with two quotations), *Taraxacum*, and *Sonchus*, reflecting frequent though less dominant usage. Rarely quoted taxa (R) comprised 29.7% (11 genera), including *Papaver*, *Urtica*, and *Silybum*. These plants may still hold ethnobotanical value, but their lower citation frequency suggests specialised, declining, or more locally restricted use.

A cross-analysis of linguistic origin and citation frequency reveals distinct cultural and ethnobotanical patterns, as shown in Table 4. The majority of taxa (23 out of 37) are associated with Greek-origin folk names, underscoring the long-standing influence of the Greek language and plant knowledge in the region. These names dominate both the Very Common (VC) and Rare (R) categories, suggesting a heritage of transmission that persists even when usage has waned. Exclusively Albanian-origin names are fewer (five out of 37), mainly appearing in the Common (C) and Rare (R) groups, indicating a localised retention of plant knowledge, particularly for widely available taxa such as *Beta*, *Pleurotus*, and *Urtica*. Hybrid Greek–Albanian names occur in nine genera, clustered in the three frequently cited categories with three genera for each one (Table 4). This clearly demonstrates a partial transformation of the traditional plant nomenclature over the past five decades.

**Figure 3:**
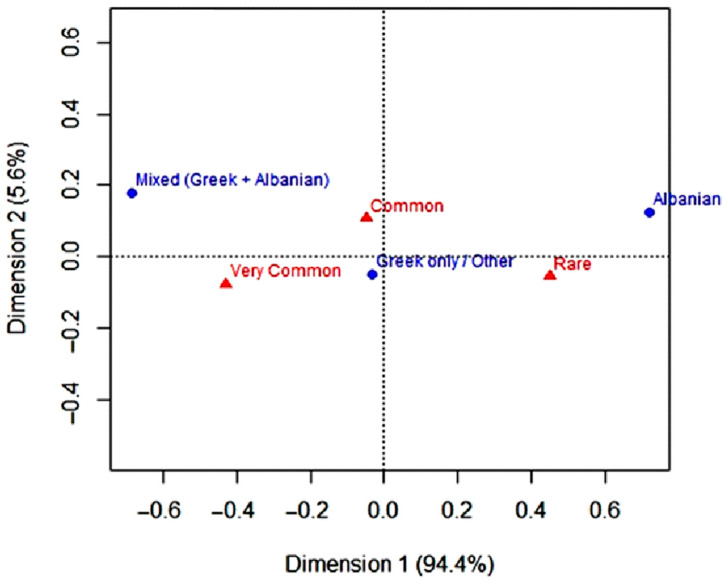
Correspondence analysis of wild green phytonyms’ linguistic origin and quotation frequency, showing clusters corresponding to the Core Resilient Heritage (high use + Albanian names) and Marginalised/Borrowed Knowledge (low use + Greek names).

The Correspondence Analysis (CA) presented in Figure 3 reveals distinct associations between the linguistic origin of plant names, their frequency of use, and the specific plant parts utilised. Plant names of Albanian origin are predominantly associated with the “Rare” and, to a lesser extent, the “Common” frequency categories, indicating that these taxa are cited or utilised less frequently within the studied communities. In contrast, Greek-origin names cluster with the “Very Common” category, suggesting broader familiarity and more widespread use.

Notably, a subset of taxa, including *Beta*, *Urtica*, and *Pleurotus*, combines relatively high citation frequency with the retention of Albanian-origin names, possibly representing a “core resilient heritage” of the Arbëreshë ethnobotanical knowledge. Conversely, taxa such as *Chondrilla*, *Notobasis*, and *Raphanus*, cited rarely and associated with Greek names, form a possibly “marginalised or newly borrowed knowledge” group. These patterns highlight that cultural resilience is unevenly distributed across the wild plant repertoire, with some species preserving linguistic and cultural continuity while others reflect assimilation and innovation.

## 4. Discussion

The comparative analysis of botanical families and plant use between the 1970 and 2025 datasets reveals significant shifts in species composition and ethnobotanical practices, reflecting broader ecological, socio-cultural, and linguistic transformations. These findings support the hypothesis that traditional ecological knowledge (TEK) and plant use are dynamic, shaped by environmental change, cultural contact, and socio-economic factors [27,28,29].

### 4.1. Botanical Diversity Change

The herbaceous wild vegetable dataset from 1970 included 21 species belonging to 21 genera across 10 families, whereas the 2025 dataset comprised 37 species from 37 genera distributed among 14 families. Seventeen genera were consistently present in both 1970 and 2025, including *Allium*, *Amaranthus*, *Centaurea*, *Cichorium*, *Origanum*, and *Taraxacum*, demonstrating continuity in core edible wild plant resources over time [30]. Meanwhile, four genera recorded in 1970 were absent in 2025. In contrast, twenty genera appeared exclusively in 2025, including *Asparagus* (Asparagaceae), *Beta* (Amaranthaceae), *Pelargonium* (Geraniaceae), and *Raphanus* (Brassicaceae), reflecting an expansion in both taxonomic diversity and ecological breadth of edible wild plants [31].

At the family level, the diversity of Asteraceae increased from eight to twelve genera, Brassicaceae from two to four, and Apiaceae from one to three, while Lamiaceae declined markedly from four to just one genus. Additionally, Fabaceae and Solanaceae, which were present in 1970, were absent from the 2025 dataset. Similar shifts in ethnobotanical records elsewhere have been linked to a combination of ecological changes, shifts in agricultural practices, and evolving cultural food preferences [32,33]. These transformations may also reflect broader socio-economic dynamics, including the decline of subsistence foraging, increased market integration, and generational changes in the transmission of plant knowledge [15].

The documented changes suggest that Arbëreshë communities, while maintaining specific emblematic bitter taxa, have also integrated new (again, predominantly bitter) edible wild species into their contemporary foodscapes, possibly due to increased ecological availability, the adaptation of traditional recipes, or the revitalisation of foraging knowledge through gastronomic and heritage movements. However, the loss of some taxa recorded in 1970, most notably the bitter *Solanum* and *Disocorea* spp. that are often markers of ancient cuisines and poverty, raises questions about the persistence of associated cultural practices and taste preferences, the resilience of local plant knowledge, and the potential erosion of biocultural diversity, also in the face of environmental and cultural (social) change [32].

### 4.2. Continuity and Transformation of Wild Greens-Centred Culinary Practices

The persistent predominance of young aerial parts in both the 1970 and 2025 datasets aligns with ethnobotanical theory, which emphasises the preference for palatable, tender, and easily harvestable plant parts in traditional diets [34,35]. Such continuity supports the idea of a profound cultural attachment to specific plant parts and preparation methods that have persisted despite environmental and socio-economic changes.

The increase in the use of flowering structures in 2025 may signal both ethnobotanical diversification and refined culinary appreciation, consistent with studies showing that changing tastes, innovation in recipes, and evolving plant knowledge can expand the range of plant parts exploited [36].

The predominance of boiled (bitter) green mixes is also reflected in the culinary preparation name, which is *lakra*, roughly meaning “wild greens” in Albanian and corresponding to the Greek term *chorta,* the South Italian term *foglia*, and the Cypriot Maronite Arabic *shkhekh*. In 2025, the relevance of these clusters of wild vegetables demonstrates remarkable cultural resilience in foraging and dietary practices, reinforcing ethnographic observations that certain culinary traditions serve as anchors of community identity across generations [37,38]. The continued use of wild plants as flavour enhancers and seasonings, as well as in preserved forms (e.g., jams, dolmades, pickling), further reflects a dynamic and adaptive food system that blends continuity with innovation [39,40]. This culinary adaptability supports the broader notion that traditional foodways are not static relics but living practices that evolve in response to ecological availability, cultural interaction, and changing socio-economic contexts [41,42].

### 4.3. Cultural Resilience and Linguistic Dynamics in Albanian Wild Greens Knowledge

Our findings raise a central question in cultural resilience studies: to what extent can resilience be claimed when embodied practices such as gathering and cooking wild greens remain active? Yet, the linguistic markers that once anchored these practices to a minority identity are lost mainly. In the case of the Arbëreshë, resilience appears to lie more in the persistence of embodied ecological knowledge than in the retention of linguistic heritage. This tension underscores the need for more comprehensive, multidimensional frameworks of resilience that encompass both external continuities and internal transformations of cultural knowledge. Our data reveal a rich and diverse use of wild greens within the Albanian community, with numerous species documented both in 1970 and 2025. This enduring ethnobotanical knowledge highlights a remarkable heritage of wild plant use, which may indicate a strong cultural resilience. However, it also raises the fundamental question of whether this tradition represents a direct continuity of the original Albanian customs brought by the community during its migration centuries ago, or whether it has been significantly influenced or even replaced by the ethnobotanical knowledge of neighbouring Greek populations. Given the strong presence of Greek-origin plant names in our dataset and the complex history of cultural contact and bilingualism in the region, it is plausible that the current repertoire results from both persistence and integration processes. Such syncretism is common in minority communities where identity, language, and traditional knowledge dynamically interact [11,43]. Understanding this balance is crucial for interpreting the cultural significance of wild greens in Arbëreshë life and their role in maintaining ethnic identity.

The significant reduction in Albanian-origin folk names, alongside the predominance of Greek-derived terminology, suggests a substantial linguistic shift likely driven by language attrition and sociopolitical pressures favouring dominant languages [11]. This shift prompts the question of whether Greek equivalents simply substituted original Albanian plant names or whether the community adopted new plant uses and names from Greek ethnobotanical traditions. Our data, showing numerous genera with mixed Greek-Albanian names and an increase in taxa only recorded in the 2025 dataset, support both processes. The replacement of names aligns with well-documented patterns of minority language erosion, where specialised vocabularies, such as plant names, are among the first to be lost or replaced [12,19]. Simultaneously, the appearance of new taxa in the recent data hints at cultural borrowing or adaptation, reflecting ongoing intercultural exchange [39]. This dual process has important implications, as it highlights the dynamic nature of traditional knowledge systems, which are not static relics but evolve in response to changing linguistic and ecological contexts [41,44].

Another possible explanation for the predominance of Greek-origin names is that the Arbëreshë may have historically drawn upon a relatively restricted repertoire of wild greens when they first settled in the area, rather than possessing a broader Albanian ethnobotanical vocabulary that was later replaced. If this were the case, the subsequent adoption of additional taxa and their corresponding Greek names could reflect an expansion of plant knowledge through long-term cultural interaction, rather than a simple linguistic substitution. This perspective highlights the challenge of distinguishing between processes of adaptation, borrowing, and continuity in minority ethnobotanical systems. It highlights the need to consider both historical depth and intercultural dynamics when interpreting the present corpus of knowledge about wild greens.

A crucial area of inquiry concerns the plants that have retained their Albanian names and uses over the decades. Two aspects are essential: (a) whether these species continue to be used similarly to their documented applications in 1970, and (b) how frequently they are cited in contemporary ethnobotanical interviews. Our preliminary analysis indicates that species such as *Sonchus*, *Beta*, and *Silene* not only preserve their Albanian names but are also commonly used in traditional recipes, signalling a sustained cultural role. Additionally, other genera with Albanian-origin names, such as *Chenopodium* and *Urtica*, although less frequently cited, still appear in interviews and hold localised importance within specific subcommunities or ecological niches. The frequency of citations serves as a proxy for cultural relevance, as documented in ethnobotanical research [44]. The active use of plants often indicates ongoing knowledge transmission and practical application. In contrast, rarely cited taxa may reflect fragmented or fading knowledge limited to older generations or particular contexts.

These taxa can be interpreted as ecological “cultural keystone species” plants that hold critical significance in shaping and maintaining cultural identity, practices, and environmental knowledge [45,46]. Such species often contribute disproportionately to community resilience and the transmission of heritage knowledge, acting as symbolic and practical anchors in times of sociolinguistic change. Their persistent use and recognition underscore their embeddedness in everyday life, culinary traditions, and cultural narratives. Identifying and prioritising these species for conservation and cultural revitalisation efforts could strengthen both biodiversity and cultural heritage in the Albanian context. Furthermore, a nuanced understanding of citation patterns across genera enables targeted strategies to support those plants and associated knowledge that are at the most significant risk of loss.

In interpreting these shifts in botanical diversity and plant use, it is essential to consider the broader historical and socioeconomic context of Arbëreshë communities. In the 1970s, many villages experienced demographic changes due to rural outmigration, shifts from subsistence agriculture to market-oriented production, and increased exposure to neighbouring Greek populations, all of which influenced foraging practices and knowledge transmission [47,48]. Historical records suggest that the Arbëreshë migration and settlement in the area occurred centuries earlier, with initial communities likely maintaining a repertoire of wild greens adapted to local ecological conditions [49,50]. Over time, interactions with Greek populations, evolving culinary preferences, and socio-economic pressures may have led to both the integration of new taxa and the replacement of Albanian-origin plant names [51,52]. These factors highlight that the current diversity of wild greens represents not only a continuation of traditional knowledge but also a product of adaptation and intercultural exchange, reflecting the dynamic interplay between ecology, culture, and livelihood practices over the last decades [53,54].

In this sense, what we refer to as “Albanian wild greens knowledge” in the studied villages should not be understood as a purely preserved and unchanged system, but rather as a layered repertoire shaped by both continuity and intercultural integration. The persistence of specific taxa with Albanian-origin names (e.g., *Sonchus*, *Beta*, *Silene*) demonstrates elements of resilience and cultural memory. In contrast, the predominance of Greek-origin plant names and the appearance of newly incorporated genera indicate processes of borrowing and replacement. Thus, the present ethnobotanical corpus is best viewed as a hybrid knowledge system, reflecting the dynamic interplay between inherited Arbëreshë practices and long-term interactions with neighbouring Greek traditions. In this light, the concepts of resilience, replacement, and integration are not contradictory but complementary in capturing the ongoing transformations of biocultural knowledge in these communities.

### 4.4. Implications for Biocultural Conservation

The intertwined dynamics of language loss and erosion of ethnobotanical knowledge underscore the urgent need for integrated biocultural conservation strategies. Language revitalisation and the preservation of traditional ecological knowledge (TEK) are mutually reinforcing processes, as linguistic diversity often embodies unique cultural perceptions of biodiversity and ecological relationships [19]. Without active efforts to maintain the Arbëreshë language, vital plant knowledge encoded in folk names, narratives, and usage practices risks disappearing, diminishing both cultural identity and environmental stewardship.

Effective conservation initiatives require a hands-on approach that bridges linguistic and ecological domains. Community-driven programmes such as bilingual education, intergenerational knowledge transmission projects, and the systematic documentation of oral histories have demonstrated success in maintaining both language and TEK [12]. For instance, in the Pacific Northwest, Indigenous language revival programmes have been paired with ethnobotanical projects that actively teach traditional uses of plants, thereby strengthening the link between linguistic and ecological knowledge [55].

Collaborative ethnobotanical research can also empower minority communities to further document, share, and revitalise their plant knowledge, and may increase the awareness among majority stakeholders of the value and richness of minority communities’ complex heritage. Examples include participatory ethnobotanical field schools, heritage-based workshops, and community-led training sessions that engage both local members and visitors, fostering awareness, pride, and continuity of traditional practices [56]. In the Mediterranean and Balkan contexts, minority groups need to further act as custodians and revitalizers of traditional ecological knowledge, integrating it with education and intercultural exchange programmes.

Such proactive, hands-on initiatives not only protect endangered linguistic and ecological knowledge but also reinforce community identity, promote environmental stewardship, and contribute to cultural resilience [57,58]. By recognising local language and ethnobotanical knowledge as complementary vessels of cultural heritage, integrated efforts can simultaneously safeguard biodiversity and the socio-cultural fabric of Arbëreshë communities, aligning with broader global frameworks that advocate for biocultural diversity as essential to sustainable development [19].

## 5. Conclusions

This study highlights a rich yet increasingly fragile repertoire of wild edible plants within the Albanian villages of the study area in central Greece, documenting 41 genera used across multiple decades. The findings underscore both the resilience and transformation of traditional ecological knowledge in the face of profound socio-cultural changes. While many species and uses documented in 1970 persist, there is evident erosion, particularly among less commonly used taxa and specialised culinary applications. Simultaneously, our data reveal an expansion of the wild plant repertoire, marked by the introduction of new species and preparations, often reflecting cultural integration and influence from neighbouring Greek communities.

The linguistic analysis reveals a marked shift in folk plant nomenclature: Based on the 2025 data, many folk plant names of Greek or mixed origin are evident, while terms of Albanian origin are less prominent. Although this distribution may reflect long-term processes of linguistic borrowing and cultural assimilation, it cannot be directly compared with the 1970 dataset, which did not record local names. It is also possible that not only names but specific uses, such as the preparation of particular wild greens, were culturally borrowed from Greek “traditions”. This lexical or cultural replacement may signal a deeper erosion among Albanians in Greece of the intimate connections between language, plant knowledge, and cultural identity.

These intertwined dynamics of language shift, changing land use patterns, and socio-economic integration into Greek society have reshaped the biocultural landscape of the Albanians. To safeguard this unique heritage, efforts must extend beyond botanical inventory toward fostering active transmission of ethnobotanical knowledge in family and community settings, coupled with initiatives to revitalise the very threatened linguistic and cultural practices. Such integrative approaches will be crucial for sustaining the rich legacy of ecological wisdom and cultural identity of Albanians in Greece for future generations.

## Figures and Tables

**Table 1:** Wild vegetables documented in the Albanian villages of the study area in Greece. The plant names in bold indicate taxa for which the recorded local names are of Greek origin; names in italics and bold indicate hybrid (Albanian and Greek) names; names rendered in plain fonts are of Albanian origin.

Botanical Taxon or Taxa	Botanical Family	Voucher Specimen Number, Starts with UVVETBOT	Local Plant Name(s)	Used Parts	Culinary Prepraration(s)	Quotation Frequency
*Allium ampeloprasum* L.	Amaryllidaceae	GRARB31	Hudhra e egër, Skordo	Whole plants	Seasoning	R
*Amaranthus blitum* L. and possibly other *Amaranthus* spp.	Amaranthaceae	GRARB27	Nena, Vlita	Tender aerial parts	Boiled	VC
*Asparagus aphyllus* L.	Asparagaceae	GRARB15	Sfaraghia, Sparaghia, Srokalià, Sporaghie, Spuraghie	Shoots	Cooked	VC
*Beta vulgaris* L.	Amaranthaceae	GRARB34	Spanakë e egër	Aerial parts	Lakra	C
*Centaurea raphanina* Sibth. & Sm.	Asteraceae	GRARB21	Aginarkai, Agriaginara, *Iannides e egër*	Young whorls	Lakra	VC
*Chenopodium album* L.	Amaranthaceae	GRARB20	Llabotë	Young aerial parts	Boiled	R
*Cichorium intybus* L.	Asteraceae		Radiki, *Radiki e kuqe*	Young aerial parts	Lakra	C
*Chondrilla juncea* L.	Asteraceae	GRARB34	Radiki	Young aerial parts	Lakra	R
*Crepis bursifolia* L.	Asteraceae	GRARB09	Prikrada, Radiki	Young aerial parts	Lakra	VC
*Crepis capillaris* (L.) Wallr.	Asteraceae		*Radiki e kuqe*	Young whorls	Lakra	VC
*Cynara cardunculus* L.	Asteraceae		Bukë e ljepuri, Iannides	Flower receptacles	Snack	C
*Eruca vesicaria* (L.) Cav.	Brassicaceae	GRARB14	Ruqë, Rukò	Young aerial parts	Salads	C
*Foeniculum vulgare* Mill.	Apiaceae	GRARB11	Anitho, Marathee, Maratho, Sidra	Aerial parts	Seasoning, Lakra	VC
*Hirschfeldia incana* (L.) Lagr.-Foss.	Brassicaceae	GRARB04	Ghinarides, Vruva	Young aerial parts	Lakra	VC
*Leontodon* spp.	Asteraceae		Glykoradiki	Young aerial parts	Lakra	C
*Malva sylvestris* L.	Malvaceae	GRARB28	Mallagë, Moaga, Molaga, Molocha	Leaves	Lakra	R
*Leopoldia comosa* (L.) Parl.	Asparagaceae		Vorvi, Vrovi, Vulvià, Vulvulli	Bulbs	Cooked, pickled	C
*Notobasis syriaca* (L.) Cass.	Asteraceae		*Glim e egër*	Stems	Snack	R
*Origanum vulgare* L.	Lamiaceae		Iasmo, Riganee	Flowering tops	Seasoning	C
*Papaver rhoeas* L.	Papaveraceae		Paparuna, Lule e kuqe	Young whorls	Lakra	R
*Pelargonium graveolens* L’Hér.	Geraniaceae		Arbarodisa	Leaves	Seasoning for jams and sweet preserves	C
*Helminthotheca echioides* (L.) Holub	Asteraceae		Radiki	Young aerial parts	Lakra	C
*Pleurotus* spp. (Fungi)	Pleurotaceae		Kepurdhë	Fruiting body	Cooked	C
*Portulaca oleracea* L.	Portulacaceae		Adrakla, Andrakla	Aerial parts	Salads	C
*Raphanus* spp.	Brassicaceae		Rapanidee	Aerial parts	Lakra	R
*Reichardia picroides* (L.) Roth	Asteraceae	GRARB05	Bukë ljepuri, Ghieres, Kochinida, Lagomomachi	Aerial parts	Salads, Lakra	VC
*Rumex* spp.	Polygonaceae		Lahana	Leaves	Lakra, dolmades	C
*Scandix pecten-veneris* L.	Apiaceae	GRARB29	Marillida, Miridis	Aerial parts	Lakra	C
*Scolymus hispanicus* L.	Asteraceae		Glim	Stems	Cooked	R
*Silene vulgaris* (Moench) Garcke	Caryophyllaceae		Bathëz	Young shoots	Lakra	C
*Silybum marianum* (L.) Gaertn.	Asteraceae	GRARB08	*Glim e gomari*	Stems	Snack	R
*Sinapis alba* L.	Brassicaceae	GRARB12	Chichibetra, Ghinarides, Piperica, Vruva, Vruvi	Young aerial parts	Lakra	VC
*Sonchus oleraceus* L.	Asteraceae	GRARB07	Rreshellë, Rreshillë, Rushellë	Young aerial parts	Lakra	VC
*Taraxacum campylodes* G.E.Haglund	Asteraceae	GRARB33	Radiki, Taraxako	Young whorls	Lakra	VC
*Tordylium apulum* L.	Apiaceae	GRARB13	Kalkafidha, Kafkalithra, Marallida, Skarkalithra	Aerial parts	Lakra	VC
*Tragopogon porrifolius* L.	Asteraceae	GRARB25	Bukë cjapi, Bukë qapi, *Brokolë e egër*	Young shoots	Salads, Lakra	C
*Urospermum* spp.	Asteraceae		Radiki	Young whorls	Lakra	R
*Urtica urens* L.	Urticaceae	GRARB19	Hith, Hithra	Leaves	Lakra	R

Lakra—Boiled green mixes.

**Table 2:** Distribution of Wild Plant Folk Names by Linguistic Origin (2025 Study).

Linguistic Origin	Number of Taxa	Percentage (%)	Genera
Albanian only	5	13.5%	*Beta*, *Chenopodium*, *Silene*, *Sonchus*, *Urtica*
Greek only	23	62.16%	*Arbutus*, *Capparis*, *Chondrilla*, *Foeniculum, Hirschfeldia*, *Leontodon*, *Muscari*, *Notobasis*, *Origanum*, *Pelargonium*, *Picris*, *Portulaca*, *Pyrus*, *Quercus*, *Raphanus*, *Rumex*, *Scandix*, *Scolymus*, *Silybum*, *Sinapis*, *Satureja*, *Taraxacum*, *Tordylium*
Hybrid (Albanian and Greek)	9	24.32%	*Amaranthus*, *Allium, Centaurea*, *Crepis, Cynara*, *Eruca*, *Malva, Papaver*, *Reichardia, Tragopogon*

**Table 3:** Quotation Frequency and Distribution of Wild Plant Genera in the 2025 Study.

Quotation Frequency	Number of Genera	Percentage (%)	Genera (List)
Common (C)	14	37.8%	*Beta*, *Cichorium*, *Cynara*, *Eruca*, *Leontodon*, *Muscari*, *Origanum*, *Pelargonium*, *Picris*, *Pleurotus*, *Portulaca*, *Rumex*, *Scandix*, *Silene*, *Tragopogon*
Very Common (VC)	12	32.4%	*Amaranthus*, *Asparagus*, *Centaurea*, *Crepis* (2), *Foeniculum*, *Hirschfeldia*, *Reichardia*, *Sinapis*, *Sonchus*, *Taraxacum*, *Tordylium*
Rare (R)	11	29.7%	*Allium*, *Chenopodium*, *Chondrilla*, *Malva*, *Notobasis*, *Papaver*, *Raphanus*, *Scolymus*, *Silybum*, *Urospermum*, *Urtica*

**Table 4:** Linguistic origin and citation frequency of the documented plant genera.

Linguistic Origin	Very Common (VC)	Common (C)	Rare (R)	Total Genera
Albanian only	*Sonchus*	*Beta*, *Silene*	*Chenopodium*, *Urtica*	5
Greek only	*Asparagus*, *Foeniculum Hirschfeldia*, *Sinapis*, *Taraxacum, Tordylium*	*Cichorium*, *Leontodon*, *Muscari*, *Origanum*, *Pelargonium, Picris*, *Portulaca*, *Rumex, Scandix*,	*Chondrilla*, *Notobasis*, *Raphanus, Scolymus*, *Silybum*, *Urospermum*	25
Hybrid (Albanian and Greek)	*Amaranthus*, *Centaurea*, *Crepis*, *Reichardia*,	*Cynara*, *Eruca*, *Tragopogon*	*Allium*, *Malva*, *Papaver*,	10
Total	12	14	11	37

## Data Availability

Data supporting the reported results are available from the corresponding author upon reasonable request.
